# 1144. Dissociative Neurological Symptom Reactions Following COVID-19 Vaccination in Adolescents in Japan

**DOI:** 10.1093/ofid/ofad500.985

**Published:** 2023-11-27

**Authors:** Yuho Horikoshi, Takayuki Yamanaka, Yuta Aizawa, Yukitsugu Nakamura, Takayo Shoji, Toshihiko Okumura, Yoshiaki Cho

**Affiliations:** Tokyo Metropolitan Children's Medical Center, Fuchu, Tokyo, Japan; Niigata City General Hospital, Niigata, Niigata, Japan; Niigata University Graduate School of Medical and Dental Sciences, Niigata, Niigata, Japan; St Marianna University School of Medicine, Kawasaki, Kanagawa, Japan; Shizuoka Children's Hospital, Shizuoka, Shizuoka, Japan; Aichi Children’s Health and Medical Center, Obu, Aichi, Japan; Okinawa Prefectural Nanbu Medical Center and Children’s Medical Center, Okinawa, Okinawa, Japan

## Abstract

**Background:**

In 2019, the WHO categorized immunization stress-related reactions as an adverse event following immunization (AEFI). Understanding AEFI is essential for determining an appropriate communication strategy to sustain trust in vaccination. Delayed response to immunization includes dissociative neurological symptom reactions (DNSRs), which are characterized by functional neurological symptoms. DNSRs following immunizations are quite rare, but it is often wrongly linked to causal relationship with vaccines that the public might perceive unnecessary fear. As DNSRs following vaccination in adolescents are currently understudied, the present report aimed to describe the clinical features of DNSRs following COVID-19 vaccination.

**Methods:**

The multicentric study was conducted between July 2021 and March 2023 at seven tertiary pediatric medical centers accepting patients with persistent AEFI. Patients aged 18 years or younger were included. Their charts were reviewed retrospectively for demographic data, symptoms, and the outcomes of DNSRs following COVID-19 vaccination.

**Results:**

In total, 46 patients aged 6 to 17 years were referred for persistent AEFI following COVID-19 vaccination. Among these, eight received the diagnosis of DNSR. Female patients comprised 87% of the cohort. All the patients had received the Comirnaty vaccine (Pfizer). Before DNSR onset, seven and one patient had received their first and second dose of Comirnaty, respectively. Two and one of the eight patients had a mental handicap and autism spectrum disorder, respectively. The DNSR symptoms included weakness (37%), gait irregularity (25%), speech difficulty (25%), paralysis (12%), and non-epileptic seizure (12%). The duration from vaccination to symptom onset ranged from 0 to 14 days (median: 1 day; IQR: 1-2 days). Symptom duration was 0.5-11 months (median: 5 months; IQR: 1-7 months). The outcomes included resolution (25%), improvement (62%) and no change (12%).

Patients with DNSR symptoms

**Figure:**
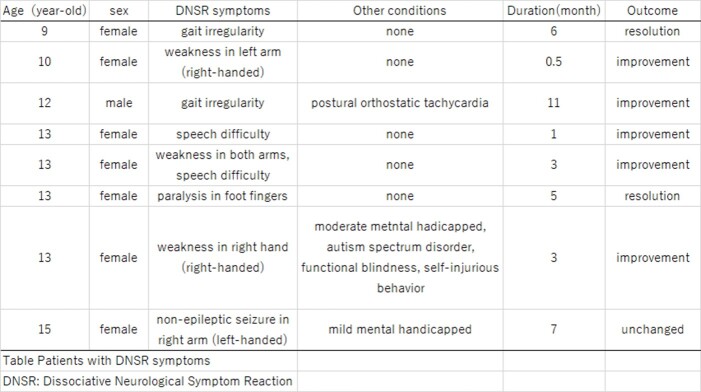

This is summary of patients presenting with DNSRs.

**Conclusion:**

DNSRs following COVID-19 vaccination were observed predominately in female, adolescent patients and had a favorable, long-term outcome.

**Disclosures:**

**Yuho Horikoshi, MD**, Shionogi Pharm: Grant/Research Support

